# Validity of the Finnish Care Register for Social Welfare in a nationwide cohort of people with Alzheimer’s disease

**DOI:** 10.1177/14034948221130150

**Published:** 2022-11-07

**Authors:** Virva Hyttinen, Kirsikka Selander, Anna-Maija Tolppanen, Riikka Väyrynen, Lasse Mielikäinen, Ismo Linnosmaa, Sirpa Hartikainen

**Affiliations:** 1 Department of Health and Social Management, University of Eastern Finland, Finland; 2 The Finnish Institute of Occupational Health, Finland; 3 School of Pharmacy, University of Eastern Finland, Finland; 4 Information and Guidance of Information Management, National Institute for Health and Welfare, Finland; 5 Kuopio Research Centre of Geriatric Care, University of Eastern Finland, Finland

**Keywords:** Care register, prescription register, validation, institutional care, social care, Alzheimer’s disease

## Abstract

**Aims:**

To assess the validity and completeness of the Care Register for Social Welfare among community-dwelling people with Alzheimer’s disease in Finland.

**Methods:**

The study was carried out in the Medication Use and Alzheimer’s disease (MEDALZ) study population, which includes 70,719 people who received a clinically verified diagnosis of Alzheimer’s disease between 2005 and 2011 and the people matched with them for comparison (*n*=282,862). The data were linked to the Care Register for Social Welfare, which contains data on care periods for nursing homes and sheltered housing with 24-h assistance during the time period 1994–2015. The validity of the Care Register for Social Welfare was analysed in relation to the Prescription Register among people with Alzheimer’s disease aged >65 years (*n*=25,640) who fulfilled the definitions of long-term care in certain inpatient care units (nursing homes, institutional care for people with dementia and rehabilitation institutions), although, in Finland, drug purchases should not be recorded in the register during long-term care.

**Results:**

The required level of assistance at discharge was recorded for 99.7% of people, diagnoses for 5.1% of the care periods and the discharge date for 100% of the completed care periods. Depending on the definition of long-term care, 6–10% of all long-term care periods included drug purchases during the study period.

**Conclusions:**

**The validity of the Care Register for Social Welfare is high, but some limitations should be considered when using the data. Combining health and social care registers provides a potentially more comprehensive database on the utilisation and costs of services.**

## Background

National administrative registers provide an attractive source of data for clinical epidemiology. Hospital discharge registers have been actively utilised in the Nordic countries and their validity has been demonstrated [1]. However, to obtain a comprehensive view of the utilisation and costs of care, data on periods of care in social institutions should also be considered. This is particularly important among service user populations consisting of vulnerable older people and people with cognitive disorders.

In many countries, inpatient hospital stays are recorded in a national hospital discharge register. Finland has one of the world’s oldest discharge registers, the Care Register for Health Care, covering the whole country [2]. It has been fully operational since 1967 and covers all inpatient hospital admissions with a personal identification code since 1969 [3]. The collection of data on the use of residential care and social care institutions began in 1996 [4]. Similar nationwide registers have also been founded in other Nordic countries [1,5,6.

The validity of the Finnish Care Register for Health Care was demonstrated earlier in a systematic review by Sund [2], which identified 32 studies validating the Care Register for Health Care and found that more than 95% of discharges could be identified from the register. The positive predictive value for several diagnoses ranged from 75 to 99%.

Although several studies have assessed the validity of hospitalisation data, to our knowledge there is still a lack of validation studies on the registers of social welfare. In Finland, the Care Register for Social Welfare contains data on care periods for nursing homes, sheltered housing with part-time or 24-h assistance, and information on the activities of institutions and their patients. The importance of validation studies has been acknowledged [7] and the validation of this specific register is further motivated by the fact that these care periods are expensive and can be responsible for a substantial proportion of the total costs in certain groups, such as people with cognitive disorders. It has been shown that costs of formal and informal care in relation to Alzheimer’s disease (AD) progression are remarkably higher in mild AD compared with early AD [8]. Validated data on service use are urgently needed due to the globally increased prevalence and incidence of cognitive disorders.

### Aims

We evaluated the validity of the Finnish Care Register for Social Welfare among people with AD, which is the most common cause of dementia [9]. The validation was performed from several perspectives, including the completeness of data and cross-checking drug purchases during long-term care from the Prescription Register. In Finland, the Prescription Register includes all outpatient drugs that are reimbursed by the Social Insurance Institution (SII) [10]. If a person is in long-term inpatient care, drugs are included in the cost of care and the SII does not reimburse drugs during these care periods [11]. People in long-term care should therefore not have drug purchases in the register during their periods of inpatient care. The validation was performed in the context of a register-based Medication Use and Alzheimer’s Disease (MEDALZ) study that includes community-dwelling people in Finland with a clinically verified diagnosis of AD and the people matched with them for comparison during the time period 2005–2011

## Materials and methods

### Identification of people with AD

The MEDALZ study includes 70,719 residents of Finland who received a clinically verified diagnosis of AD during the time period 2005–2011 and were community-dwelling at the time of diagnosis, as well as the 282,862 people without AD matched with them for age, sex and region of residence. People with AD were identified from the Special Reimbursement Register maintained by the SII [12]. On the date of diagnosis with AD, each person with AD was matched with up to four people for comparison, identified from the SII database covering all residents using incidence density sampling. The matching criteria were as follows: same sex; residence in the same hospital district on the index date; and same age (+/− one year) on the index date. In both groups, the average age was 80 years and 65.2% were women. Supplemental Table 1 gives the descriptive statistics for people with AD and the comparison people without AD according to age, sex and university hospital district area.

The diagnosis of AD in the register is based on the reimbursement of anti-dementia drugs, which are a recommended treatment for all people with AD according to the Finnish Current Care Guideline if there are no contraindications [13]. For the reimbursement, the diagnosis of AD should be clinically verified according to the criteria defined by the Alzheimer’s Association (known earlier as the National Institute of Neurological and Communicative Disorders and Stroke and the Alzheimer’s Disease and Related Disorders Association) and The Diagnostic and Statistical Manual of Mental Disorders (DSM-IV) [14,15]. For a diagnosis of AD to be verified by the SII, a person needs to fulfill all the following criteria: (a) symptoms consistent with AD; (b) a decrease in social capacity over a period of at least three months; (c) have received a computed tomography/magnetic resonance imaging scan; (d) possible alternative diagnoses excluded; and (e) received confirmation of the diagnosis by a registered geriatrician or neurologist.

### Care Register for Social Welfare

The MEDALZ data were combined with the Care Register for Social Welfare, which is a national client register maintained by the National Institute for Health and Welfare. This register contains data on care periods for nursing homes and sheltered housing with 24-h assistance during the time period 1994–2015. In addition to admission and discharge dates, the register includes information on the required level of assistance, diagnosis (the World Health Organization’s International Classification of Diseases, 10th Revision coding system [16]), the main reasons for being institutionalised (e.g. the client’s inability to take care of him/herself or a carer’s vacation) and information on the service provider.

### Inpatient and outpatient care

Municipalities, the basic providers of welfare services in Finland, can arrange health and social services as in- or outpatient care for older people who are not able to live independently in their own homes (Supplemental Table 2). According to the decree given by the Ministry of Social Affairs and Health (1806/2009), inpatient social care includes care in nursing homes and other social care institutions providing care, rehabilitation and maintenance care, such as institutions for people with intellectual disability and substance abuse. Inpatient social care means care for people who do not need hospital care, but do not manage at home or in any other outpatient care setting, even with the help of regular social and health care services [17]. Outpatient social care is defined as care provided in a home or home-like setting, such as home care or care provided in sheltered housing units.

### Prescription Register

To evaluate whether people in long-term care had recorded drug purchases in the Prescription Register during their inpatient care periods, the Prescription Register maintained by the SII was linked with the Care Register for Social Welfare using the personal identification codes. The registers were combined from 1994 to 2015. These analyses were limited to 25,640 people aged >65 years on the first day of the care period, admitted to nursing homes, institutional care for people with dementia or rehabilitation institutions, and whose care period met our definition of long-term care. This restriction was used because the majority of people with AD are older people and most of the care periods that met our definition of long-term care were in these types of care unit (Supplemental Table 3). If the person fulfills the definition of long-term care and the information on inpatient care from the Care Register for Social Welfare is valid, then there should be no drug purchases during the inpatient care periods. We also linked information on long-term care decisions from the SII to the data.

### Validity analysis

Our validity analysis of the Care Register for Social Welfare consisted of three steps. First, we checked possible human errors and removed care periods if the discharge date was recorded before the admission date. In addition, we checked the data for duplicate care periods, indicating that the same person had more than one admission to the same service provider on the same admission day. Before counting the duplicate care periods, we imputed possible missing information on diagnosis and the required level of assistance at discharge from the other duplicate records and only one of the duplicate observations was kept in the further analyses. Before that, we removed records indicating end-of-year patient counts (carried out annually on 31 December), which are care periods that continued after the turn of the year. These were identified by a separate field indicating the type of records and were removed only if they had the same admission date and service provider as those care periods that were recorded as a completed care period.

Second, we evaluated the completeness of recording in the following fields of the Care Register for Social Welfare: discharge dates; diagnosis information; and information about the required level of assistance at discharge. We evaluated whether the missing discharge dates could be imputed with the date of death (obtained from causes of death data from Statistics Finland (2005–2012)) because some of the missing discharge dates could have been due to forgetting to report the person as discharged after death. The imputation was made if the person had a date of death and the current care period was the last one. In addition, we analysed whether missing information for discharge dates, diagnosis and required level of assistance at discharge varied over time or between the service providers.

Third, we analysed the validity of the Care Register for Social Welfare in relation to the Prescription Register by detecting whether people with a long-term care decision had drug purchases. We evaluated which proportion of care periods included drug purchases in different years and service providers. All statistical analyses were performed using Stata software (version 15.1; StataCorp LLC, College Station, TX, USA).

### Definitions of long-term care

The following three separate definitions of long-term care were used: (a) information from the Care Register for Social Welfare on whether the person had a long-term care decision (yes/no); (b) information from the SII on whether the person had a long-term care decision (yes/no).; and (c) information from the Care Register for Social Welfare on whether the person had been in the current care unit for more than 90 days. All definitions were also combined so that a person was defined to have long-term care if (s)he fulfilled at least one of these three criteria.

### Ethical issues

This study fulfilled all Finnish ethical standards. According to Finnish legislation, there was no need for ethics committee approval because the data were pseudonymised prior to being delivered to the researchers. Appropriate permissions to access the data used in this study were obtained from each register maintainer (National Institute for Health and Welfare, SII and Statistics Finland).

## Results

A total of 1,001,010 care periods for 138,673 people in the MEDALZ study were identified from the Care Register for Social Welfare (Supplemental Figure 1). The date of admission and service provider were recorded for all periods. We removed care periods with a discharge date before the admission date (*n*=3) and duplicate recording of continuing care periods from the end-of-year patient count on 31 December if these had the same admission date and same service provider as the completed care periods (*n*=213,981). After these removals, there were 5329 (0.5%) duplicate care periods (the same person had been enrolled on the same admission day more than once for the same service provider). The least informative duplicative record(s) were removed from the validity analyses, resulting in 781,697 unique care periods (Supplemental Figure 1).

Discharge dates were recorded for all (100%) of the completed care periods (695,762). When we also included unique care periods from the end-of-year patient count that were not later recorded as completed, discharge dates were recorded for 89.5% (699,424) of the care periods (Table I). There were more missing discharge dates at the start of the study period than in more recent years. Of all the missing discharge dates, more than 11% could be imputed by the date of death. After the imputation, most of the care periods with missing discharge dates were still at the start of the study period. An estimate of the required level of assistance at discharge was recorded for 99.7% (779,417) and diagnosis information for 5.1% (40,113) of the care periods.

**Table I.** Validity analysis.

Year	*N*	Missing discharge date^a^	Missing discharge dates after imputation with the date of death	Missing diagnosis	Missing amount of required level of assistance at discharge
1994	1887	1416 (75.0)	1294 (68.6)	1739 (92.2)	16 (0.9)
1995	1504	601 (40.0)	534 (35.5)	1478 (98.3)	6 (0.4)
1996	1514	496 (32.8)	439 (29.0)	1235 (81.6)	11 (0.7)
1997	1844	476 (25.8)	438 (23.5)	1577 (85.5)	67 (3.6)
1998	2503	653 (26.1)	589 (23.5)	2204 (88.1)	31 (1.2)
1999	3612	732 (20.3)	648 (17.9)	3213 (89.0)	51 (1.4)
2000	4496	936 (20.8)	848 (18.8)	3998 (88.9)	64 (1.4)
2001	5333	1178 (22.1)	1037 (19.4)	4859 (91.1)	168 (3.2)
2002	7532	1249 (16.6)	1055 (14.1)	6885 (91.4)	49 (0.7)
2003	9817	1602 (16.3)	1412 (14.4)	9099 (92.7)	87 (0.9)
2004	13,985	2128 (15.2)	1804 (12.8)	13,025 (93.1)	48 (0.3)
2005	20,411	2592 (12.7)	2097 (10.3)	19,143 (93.8)	29 (0.1)
2006	29,791	3172 (10.7)	2592 (8.7)	28,180 (94.6)	78 (0.3)
2007	42,191	4285 (10.2)	3448 (8.2)	39,776 (94.3)	274 (0.7)
2008	54,584	5339 (9.8)	4379 (8.0)	51,587 (94.5)	451 (0.8)
2009	66,496	5481 (8.2)	4359 (6.5)	63,240 (95.1)	265 (0.4)
2010	77,048	6110 (7.9)	4826 (6.3)	73,356 (95.2)	9 (<0.01)
2011	86,894	6466 (7.4)	5298 (6.1)	83,194 (95.7)	119 (0.1)
2012	90,752	6474 (7.1)	5526 (6.1)	85,923 (94.7)	235 (0.3)
2013	90,326	7255 (8.0)	6625 (7.3)	84,896 (94.0)	195 (0.2)
2014	84,870	8967 (10.6)	8670 (10.2)	80,583 (95.0)	8 (0.01)
2015	84,307	14,665 (17.4)	14,664 (17.4)	82,394 (97.7)	19 (0.02)
Total	781,697	82,273 (10.5)	72,582 (9.3)	741,584 (94.9)	2,280 (0.3)

Data presented as frequency (%) for each year.

a Periods of continuing care.

When the extent of missing information was assessed on the service provider levels, most of the missing discharge dates were in day care hospitals and in housing with part-time assistance for people with intellectual disabilities, with 85 and 77% of the care periods after imputation with the date of death, respectively (Table II). Most of the missing discharge dates could be imputed in sheltered housing (17% of missing discharge dates) and in day care hospitals (15%). The completeness of the recording of the required level of assistance at discharge showed considerable variation between service providers. In the institutions for substance abusers, the required level of assistance was recorded less often (for 5% of care periods) than in other types of service provider. Diagnosis information was most often recorded in institutions providing care for people with intellectual disability (27.3%), substance abuse (21.6%) and dementia (15.8%).

**Table II.** Validity analysis.

Type of care	*N*	Missing discharge date^a^	Missing discharge dates after imputation with the date of death	Missing diagnosis	Missing amount of required level of assistance at discharge
*Inpatient care*					
Nursing homes	302,855	10,021 (3.3)	8874 (2.9)	282,769 (93.4)	1083 (0.4)
Institutions for people with intellectual disability	930	48 (5.2)	47 (5.1)	676 (72.7)	0 (0.0)
Institutions for substance abusers	3677	24 (0.7)	23 (0.6)	2884 (78.4)	187 (5.1)
Institutional care for people with dementia	12,659	751 (5.9)	666 (5.3)	10,657 (84.2)	15 (0.1)
Rehabilitation institutions	21,636	136 (0.6)	118 (0.6)	20,352 (94.1)	0 (0.0)
Total number of inpatients	341,757	10,980 (3.2)	9728 (2.8)	317,338 (92.9)	1285 (0.4)
*Outpatient care*					
Sheltered housing with 24-h assistance for older people	330,665	38,201 (11.6)	34,506 (10.4)	319,136 (96.5)	473 (0.1)
Intensive 24-h care for people with dementia	66,184	10,202 (15.4)	9086 (13.7)	63,703 (96.3)	76 (0.1)
Housing with 24-h assistance for people with intellectual disabilities	2981	543 (18.2)	512 (17.2)	2824 (94.7)	9 (0.3)
Housing with part-time assistance for people with intellectual disabilities	456	354 (77.6)	350 (76.8)	419 (91.9)	2 (0.4)
Supportive housing for people with intellectual disability	110	84 (76.4)	80 (72.7)	102 (92.7)	0 (0.0)
Sheltered housing	31,041	20,548 (66.2)	17,062 (55.0)	30,404 (98.0)	324 (1.0)
Sheltered housing for people with psychiatric disorders	671	437 (65.1)	409 (61.0)	578 (86.1)	1 (0.2)
Sheltered housing with 24-h assistance	947	128 (13.5)	115 (12.1)	886 (93.6)	5 (0.5)
Sheltered housing with 24-h assistance for people with psychiatric disorders	1549	321 (20.7)	304 (19.6)	1452 (93.7)	1 (0.1)
Day care hospital	78	78 (100.0)	66 (84.6)	78 (100.0)	0 (0.0)
Total outpatient	434,682	70,901 (16.3)	62,490 (14.4)	419,582 (96.5)	891 (0.2)
Missing information of the care unit	5258	397 (7.6)	364 (6.9)	4664 (88.7)	104 (2.0)
Overall total	781,697	82,273 (10.5)	72,582 (9.3)	741,584 (94.9)	2280 (0.3)

Data presented as frequency (%) for each year.

a Periods of continuing care.

### Drug purchases during long-term inpatient care

**Figure 1.**
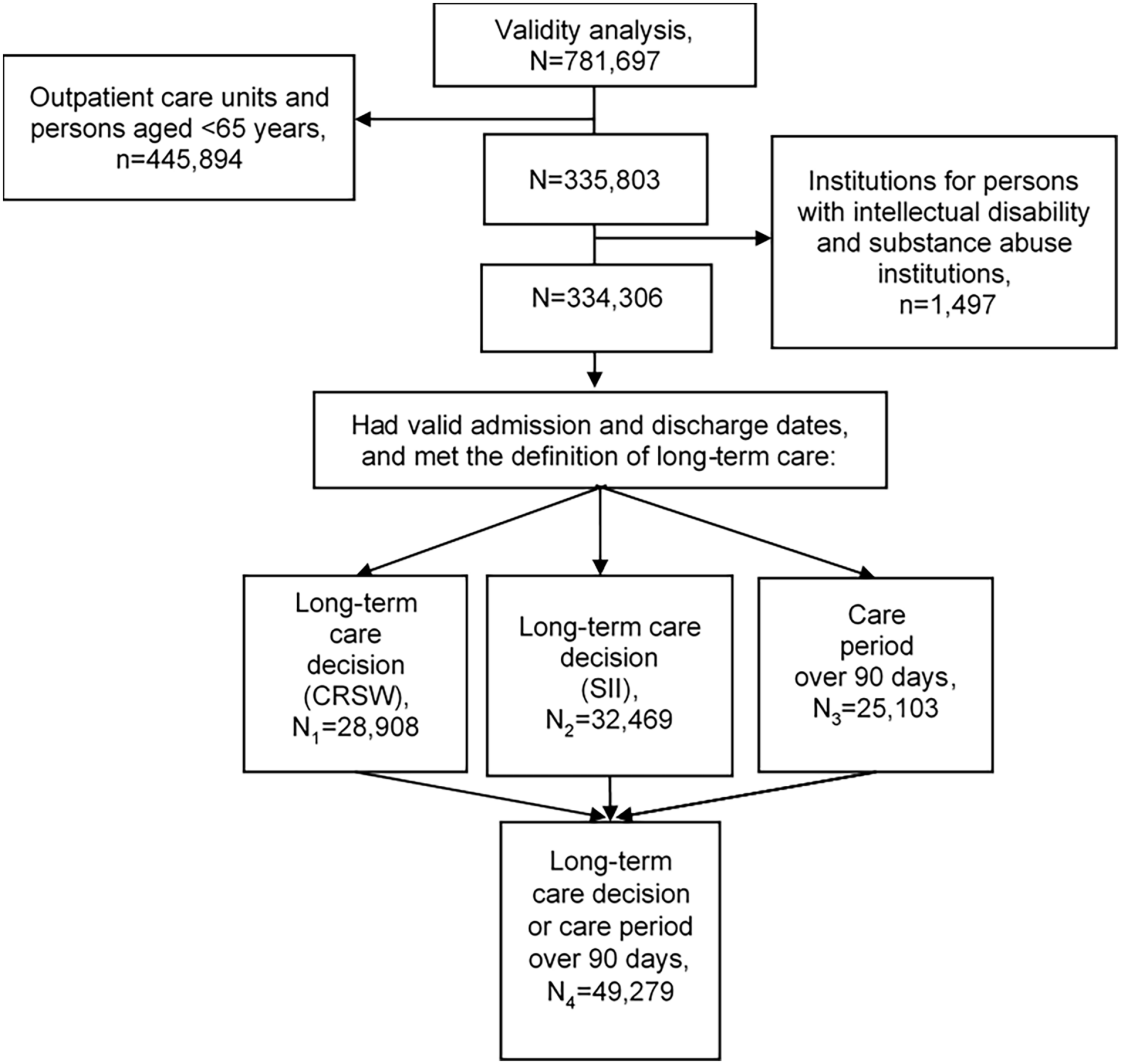
Flowchart of dataset derivation for assessment of the cross-check analysis of the Prescription Register. CRSW, Care Register for Social Welfare; SII, Social Insurance Institution.

The number of care periods fulfilling the definition of long-term care varied depending on the definition. A total of 28,908 care periods had a long-term care decision recorded in the Care Register for Social Welfare (N_1_), information about the long-term care decision from the SII was identified for 32,469 care periods (N_2_) and 25,103 care periods had lasted more than 90 days (N_3_). A total of 49,279 care periods fulfilled at least one of these definitions (N_4_) (Figure 1 and Supplemental Tables 3 and 4).

In total, 6–10% of care periods with a definition of long-term care included drug purchases during the study period (1994–2015) (Table III). The percentage of long-term care periods with drug purchases was higher in the earlier half of the study period, although the number of people with a long-term care decision was very small. Drug purchases during long-term care were common in rehabilitation institutions and less common in nursing homes or institutional care for people with dementia (Table IV). Imputation of missing discharge dates by date of death did not decrease the proportion of care periods with drug purchases.

**Table III.** Drug purchases during the care periods using definitions of long-term care.

Year	Long-term care decision (CRSW)	Long-term care decision (SII)	Care period >90 days	Long-term care decision or care period >90 days
1994	5 (71.4)	1 (25.0)	5 (62.5)	5 (62.5)
1995	2 (40.0)	0 (0.0)	3 (50.0)	3 (50.0)
1996	1 (100.0)	1 (25.0)	4 (57.1)	4 (57.1)
1997	3 (37.5)	2 (33.3)	5 (55.6)	5 (45.5)
1998	9 (45.0)	0 (0.0)	11 (42.3)	11 (39.3)
1999	2 (6.7)	2 (5.3)	13 (28.3)	15 (28.3)
2000	20 (16.7)	11 (6.7)	40 (33.6)	49 (22.7)
2001	21 (15.7)	29 (13.5)	58 (49.2)	75 (26.8)
2002	16 (11.1)	21 (9.3)	53 (38.1)	65 (20.3)
2003	15 (7.9)	24 (9.0)	64 (38.8)	79 (20.2)
2004	26 (12.8)	26 (8.6)	46 (30.3)	68 (14.9)
2005	42 (12.8)	42 (8.0)	68 (23.6)	106 (15.0)
2006	78 (8.0)	150 (8.3)	121 (14.4)	241 (12.0)
2007	88 (5.3)	208 (6.6)	160 (10.2)	296 (8.7)
2008	122 (5.7)	296 (7.1)	184 (9.2)	373 (8.5)
2009	162 (5.1)	350 (6.2)	260 (8.9)	470 (7.8)
2010	201 (5.1)	321 (6.6)	267 (7.7)	519 (7.8)
2011	262 (6.3)	240 (6.2)	363 (9.6)	558 (8.4)
2012	265 (6.6)	201 (7.2)	357 (10.0)	531 (8.7)
2013	172 (4.9)	170 (7.6)	196 (6.7)	388 (7.4)
2014	90 (3.4)	96 (7.5)	139 (6.8)	258 (6.6)
2015	63 (4.3)	91 (11.0)	94 (10.3)	210 (9.0)
Total	1665 (5.8)	2282 (7.0)	2511 (10.0)	4329 (8.8)

Data presented as frequency (%) of how many of those with long-term care decision had drug purchases for each year. Analysis limited to people aged >65 years being treated in nursing home, institutional care for people with dementia or in a rehabilitation institution (and had valid admission and discharge dates) and to those care periods that met our definition of long-term care (Supplemental Table 4).

CRSW, Care Register for Social Welfare; SII, Social Insurance Institution.

**Table IV.** Drug purchases during the care periods using definitions of long-term care for each service provider.

Type of care	Long-term care decision (CRSW)	Long-term care decision (SII)	Care period >90 days	Long-term care decision or care period >90 days
Nursing homes	1541/26,782 (5.8)	2106/30,248 (7.0)	2258/23,192 (9.7)	3949/45,806 (8.6)
Institutional care for people with dementia	85/1783 (4.7)	129/1948 (6.6)	183/1702 (10.8)	251/2857 (8.8)
Rehabilitation institutions	39/343 (11.4)	47/273 (17.2)	70/209 (33.5)	129/616 (21.0)

Data presented as frequency (%) of how many of those with long-term care decision had drug purchases for each year. Analysis is limited to people >65 years who had valid admission and discharge dates and to those care periods that met our definition of long-term care (Supplemental Table 3).

CRSW, Care Register for Social Welfare; SII, Social Insurance Institution.

## Discussion

The Finnish Care Register for Social Welfare provides a unique opportunity to examine the validity of register-based information on the use of social care services. This study showed that 99.7% of estimates of the required level of assistance at discharge and all (100%) discharge dates for completed care periods were recorded in the Care Register for Social Welfare in the context of the register-based MEDALZ study including community-dwelling people with AD in 2005–2011 and the people matched with them for comparison. However, diagnosis information was missing for 94.9% of the care periods.

To our knowledge, this is the first study evaluating the validity of the Care Register for Social Welfare. Although the discharge dates for all completed care periods were recorded in the register, it should be noted that for care periods that continued after the turn of the year, the discharge dates were missing from 10.5% of care periods, even if the person had died. This can be partially corrected by imputation with the date of death, especially in care periods at the beginning of the study period. Our results are in line with those of an earlier review that evaluated the validity of the hospital discharge register (currently a part of the Care Register for Health Care) and reported that more than 95% of discharges could be identified from the register [2]. In Denmark, the validity of Statistics Denmark’s nursing home register has been studied by comparing the information about whether or not a person is a permanent nursing home resident with administrative municipality records, which identify all individuals living in nursing homes [18]. The Danish study reported that the overall positive predictive value of the Statistics Denmark register was low (53%), but there was wide variation between municipalities (from 51 to 96%).

We found that the information about the required level of assistance at discharge was recorded for nearly all care periods. This information was missing only for 0.3% of care periods and the completeness was over 99% during the most recent years. The required level of assistance reflects the functional disabilities of residents and patients, which is needed for planning personnel resources in these units and probably explains the high coverage of this field in the register. In addition, the information could be used in social services research. However, only 5% of diagnoses were recorded in the register. This is considerably lower than, for example, the national hospital discharge patient register. The completeness of diagnostic information in nationwide hospital discharge patient registers was evaluated to be high, especially for those patients who require hospitalisation [19].

Our analysis of the validity of the Care Register for Social Welfare in relation to the Prescription Register showed that drug purchases were recorded during 6–10% (depending on the definition of long-term care) of all care periods during the study period, even if the observations fulfilled the definition of long-term care. The percentage of drug purchases during long-term care was higher at the start of the study period, so the validity of the long-term care decision was better in more recent years. However, the number of people to whom any of the definitions of long-term care have applied in this study was much smaller at the start of the study period. It is therefore important to acknowledge that the validity of the long-term care decisions or definitions may be compromised.

The strength of this study is the extensive nationwide individual-level data available to study the validation of register-based information of social care services for over two decades (1994–2015). This study provides valuable information for the application of register-based data to different purposes – for example, to research planning of social services. The confirmation of purchased prescriptions during long-term care was performed only for people with AD, so the generalizability of the results may be limited. In addition, the definitions of long-term care used in this study have not been validated, although they included the SII decision on long-term care. To increase the robustness of the results, we used three different definitions for long-term care. One potential limitation could also be that we did not perform the cross-check analysis for all service providers. However, our approach is justified by the fact that the chosen service providers are those most commonly used among people aged >65 years.

## Conclusions

This validation study showed that the Care Register for Social Welfare had high validity in relation information completeness for the required level of assistance at discharge or discharge dates of completed care periods. Only 6–10% of all long-term care periods included drug purchases during the study period. The combined health and social care registers provide a potentially more comprehensive database on the utilisation and costs of services. When utilising register-based data in research, it is still important to bear in mind register-specific limitations and the original administrative purpose of the registers.

## Supplemental Material

sj-docx-1-sjp-10.1177_14034948221130150 – Supplemental material for Validity of the Finnish Care Register for Social Welfare in a nationwide cohort of people with Alzheimer’s disease Supplemental material, sj-docx-1-sjp-10.1177_14034948221130150 for Validity of the Finnish Care Register for Social Welfare in a nationwide cohort of people with Alzheimer’s disease by Virva Hyttinen, Kirsikka Selander, Anna-Maija Tolppanen, Riikka Väyrynen, Lasse Mielikäinen, Ismo Linnosmaa and Sirpa Hartikainen in Scandinavian Journal of Public Health

sj-docx-2-sjp-10.1177_14034948221130150 – Supplemental material for Validity of the Finnish Care Register for Social Welfare in a nationwide cohort of people with Alzheimer’s disease Supplemental material, sj-docx-2-sjp-10.1177_14034948221130150 for Validity of the Finnish Care Register for Social Welfare in a nationwide cohort of people with Alzheimer’s disease by Virva Hyttinen, Kirsikka Selander, Anna-Maija Tolppanen, Riikka Väyrynen, Lasse Mielikäinen, Ismo Linnosmaa and Sirpa Hartikainen in Scandinavian Journal of Public Health
