# Supplementary material for: Validity of the Finnish Care Register for Social Welfare in a nationwide cohort of people with Alzheimer’s disease
Source: Scand J Public Health. 2022 Nov 7;52(2):136–44. doi: 10.1177/14034948221130150 (PMC10913286; doi:10.1177/14034948221130150)
Supplement: sj-docx-1-sjp-10.1177_14034948221130150 – Supplemental material for Validity of the Finnish Care Register for Social Welfare in a nationwide cohort of people with Alzheimer’s disease [file sj-docx-1-sjp-10.1177_14034948221130150.docx]

**Supplement Figures**

**Supplement Figure 1.** Flowchart of dataset derivation for assessment of the recording coverage
