# Supplementary material for: Validity of the Finnish Care Register for Social Welfare in a nationwide cohort of people with Alzheimer’s disease
Source: Scand J Public Health. 2022 Nov 7;52(2):136–44. doi: 10.1177/14034948221130150 (PMC10913286; doi:10.1177/14034948221130150)
Supplement: sj-docx-2-sjp-10.1177_14034948221130150 – Supplemental material for Validity of the Finnish Care Register for Social Welfare in a nationwide cohort of people with Alzheimer’s disease [file sj-docx-2-sjp-10.1177_14034948221130150.docx]

**Supplement Tables**

**Supplement Table 1.** Descriptive statistics for MEDALZ cohort.

|  | Persons with AD (n=70,719) | Comparison persons without AD (n=282,862) |
| --- | --- | --- |
| Age^1^, mean (SD) | 80.1 (7.1) | 80.0 (7.1) |
| Gender, n (%) |  |  |
| Women | 46,117 (65.2) | 184,463 (65.2) |
| Men | 24,602 (34.8) | 98,399 (34.8) |
| University hospital district^2^, n (%) |  |  |
| HYKS | 19,603 (27.7) | 78,412 (27.7) |
| KYS | 14,629 (20.7) | 58,516 (20.7) |
| OYS | 10,157 (14.4) | 40,625 (14.4) |
| TAYS | 15,494 (21.9) | 61,965 (21.9) |
| TYKS | 10,648 (15.1) | 42,592 (15.1) |
| Åland | 188 (0.3) | 752 (0.3) |
| AD diagnosis year, n (%) |  |  |
| 2005 | 8,547 (12.1) | *NA* |
| 2006 | 8,803 (12.5) | *NA* |
| 2007 | 9,442 (13.4) | *NA* |
| 2008 | 10,327 (14.6) | *NA* |
| 2009 | 10,500 (14.9) | *NA* |
| 2010 | 10,878 (15.4) | *NA* |
| 2011 | 12,222 (17.3) | *NA* |
| AD, Alzheimer’s diagnosis; SD, standard deviation; HYKS, Helsinki University district area; KYS, Kuopio University Hospital district area; OYS, Oulu University Hospital district area; TAYS, Tampere University Hospital district area; TYKS, Turku University Hospital district area  ^1^At the index date (the date of AD diagnosis and the corresponding matching date for comparison persons) | | |

**Supplement Table 2.** Based on the Act 1806/2009, service providers can be classified as in- or outpatient care

| Inpatient care | Outpatient care |
| --- | --- |
| Nursing homes and other care for older persons under the Social Welfare act | Sheltered housing with 24-hour assistance for older persons: assisted living in a housing unit for older persons, where staff is available around the clock |
| Institutional care for persons with dementia: care provided due to dementia | Intensive 24-hour care for persons with dementia: care provided due to dementia in units where staff is available around the clock |
| Institutions for persons with intellectual disability: care provided due to intellectual disability in a central institution or other inpatient care unit for persons with intellectual disability | Housing with 24-hour assistance for people with intellectual disabilities: housing services for persons with intellectual disability in a group home where staff is available around the clock |
| Institutions for substance abusers: care for substance abuse in a clinical treatment unit or in a substance abuse rehabilitation unit that has been approved by SII as eligible for rehabilitation funding | Housing with part-time assistance for people with intellectual disabilities: housing services for persons with intellectual disability in a group home where staff is available only part of the day |
| Rehabilitation institutions: 24-hour care in a unit where, in addition to basic care, the client receives rehabilitation services. These can include rehabilitation research, therapy, rehabilitation guidance, adaptation training in other capacity-building coaching, and other assistive services. This category does not include substance abuse institutions. | Supportive housing for persons with intellectual disability: persons with intellectual disability living in their own flat (rented or owned), with the support of a support person a few hours a week |
|  | Sheltered housing: housing services primarily for older persons and disabled persons in a unit where staff is available part of the day |
|  | Sheltered housing for persons with psychiatric disorders: housing service for persons with mental health problems in a unit where staff is available only part of the day |
|  | Sheltered housing with 24-hour assistance: housing services primarily for severely disabled persons in a unit where staff is available around the clock. This category does not include persons with intellectual disability. |
|  | Sheltered housing for persons with psychiatric disorders with 24-hour assistance: housing services for persons with mental health problems in a unit where staff is available around the clock and the main goal of living is learning everyday skills and how to survive with the disease. |
|  | Day care hospital |

**Source:** HILMO 2019, p. 36‒38.

**Supplement Table 3.** Three definitions of long-term care. The table shows how many care periods provided by each service provider met the definition of long-term care. Frequencies (n) and percentages (%) are presented. Only bolded care periods are included in the drug purchase analysis

|  | N | Long-term care decision (CRSW), n (%) | Long-term care decision (SII),  n (%) | Care period  over 90 days,  n (%) | Long-term care decision or care period over 90 days,  n (%) |
| --- | --- | --- | --- | --- | --- |
| **Nursing homes** | **290,346** | **26,782 (9.2)** | **30,248 (10.4)** | **23,192 (8.0)** | **45,806 (15.8)** |
| Institutions for persons with intellectual disability | 158 | 37 (23.4) | 25 (15.8) | 26 (16.5) | 42 (26.6) |
| Substance abuse institutions | 3,259 | 4 (0.3) | 16 (1.2) | 16 (1.2) | 32 (2.4) |
| **Institutional care for persons with dementia** | **11,797** | **1,783 (15.1)** | **1,948 (16.5)** | **1,702 (14.4)** | **2,857 (24.2)** |
| **Rehabilitation institutions** | **21,321** | **343 (1.6)** | **273 (1.3)** | **209 (1.0)** | **616 (2.9)** |
| Total | 324,939 | 28,949 (8.9) | 32,510 (10.0) | 25,145 (7.7) | 49,353(15.2) |

CRSW, Care Register for Social Welfare; SII, Social Insurance Institution

**Note:** Analysis is limited to persons aged >65 years and who had valid admission and discharge dates.

**Supplement Table 4.** Three definitions of long-term care. The table shows how many care periods met the definition of long-term care in each year. Frequencies (n) and percentages (%) are presented.

|  | N | Long-term care decision (CRSW),  n (%) | Long-term care decision (SII), n  (%) | Care period over 90 days,  n (%) | Long-term care decision or care period over 90 days,  n (%) |
| --- | --- | --- | --- | --- | --- |
| 1994 | 11 | 7 (63.6) | 4 (36.4) | 8 (72.7) | 8 (72.7) |
| 1995 | 77 | 5 (6.5) | 1 (1.3) | 6 (7.8) | 6 (7.8) |
| 1996 | 11 | 1 (9.1) | 4 (36.4) | 7 (63.6) | 7 (63.6) |
| 1997 | 33 | 8 (24.2) | 6 (18.2) | 9 (27.3) | 11 (33.3) |
| 1998 | 55 | 20 (36.4) | 16 (29.1) | 26 (47.3) | 28 (50.9) |
| 1999 | 684 | 30 (4.4) | 38 (5.6) | 46 (6.7) | 53 (7.8) |
| 2000 | 2,464 | 120 (4.9) | 165 (6.7) | 119 (4.8) | 216 (8.8) |
| 2001 | 2,666 | 134 (5.0) | 215 (8.1) | 118 (4.4) | 280 (10.5) |
| 2002 | 4,232 | 144 (3.4) | 225 (5.3) | 139 (3.3) | 321 (7.6) |
| 2003 | 5,249 | 189 (3.6) | 266 (5.1) | 165 (3.1) | 391 (7.5) |
| 2004 | 7,424 | 204 (2.8) | 304 (4.1) | 152 (2.1) | 457 (6.2) |
| 2005 | 11,324 | 328 (2.9) | 528 (4.7) | 288 (2.5) | 706 (6.2) |
| 2006 | 16,951 | 975 (5.8) | 1,819 (10.7) | 839 (5.0) | 2,015 (11.9) |
| 2007 | 22,561 | 1,654 (7.3) | 3,158 (14.0) | 1,564 (6.9) | 3,393 (15.0) |
| 2008 | 27,582 | 2,160 (7.8) | 4,160 (15.1) | 2,005 (7.3) | 4,388 (15.9) |
| 2009 | 33,191 | 3,183 (9.6) | 5,642 (17.0) | 2,921 (8.8) | 6,038 (18.2) |
| 2010 | 33,880 | 3,973 (11.7) | 4,894 (14.5) | 3,470 (10.2) | 6,670 (19.7) |
| 2011 | 35,941 | 4,158 (11.6) | 3,886 (10.8) | 3,788 (10.5) | 6,653 (18.5) |
| 2012 | 35,372 | 4,034 (11.4) | 2,787 (7.9) | 3,558 (10.1) | 6,121 (17.3) |
| 2013 | 32,273 | 3,500 (10.8) | 2,242 (7.0) | 2,933 (9.1) | 5,239 (16.2) |
| 2014 | 26,947 | 2,620 (9.7) | 1,279 (4.8) | 2,033 (7.5) | 3,934 (14.6) |
| 2015 | 24,536 | 1,461 (6.0) | 830 (3.4) | 909 (3.7) | 2,344 (9.6) |
| Total | 323,464 | 28,908 (8.9) | 32,469 (10.0) | 25,103 (7.8) | 49,279 (15.2) |

CRSW, Care Register for Social Welfare; SII, Social Insurance Institution

**Note:** The table includes only persons aged >65 years and who were treated

in nursing home, institutional care for persons with dementia,

or in a rehabilitation institution (and had valid admission and discharge dates).
